# The influence of three diphenylpyran isomer co-sensitizers with different sterical structures on N719-based dye sensitized solar cells†

**DOI:** 10.1039/d0ra08276g

**Published:** 2020-12-07

**Authors:** Xinxin Wang, Xiuhua Hang, Altan Bolag, Wu Yun, Tana Bao, Jun Ning, Hexig Alata, Tegus Ojiyed

**Affiliations:** Inner Mongolia Key Laboratory for Physics and Chemistry of Functional Materials, College of Physics and Electronic Information, Inner Mongolia Normal University No 81 Zhaowuda Road, Saihan District Hohhot 010022 China altan.bolag@imnu.edu.cn; Inner Mongolia Key Laboratory for Environmental Chemistry, College of Chemistry and Environmental Science Inner Mongolia Normal University No 81 Zhaowuda Road, Saihan District Hohhot 010022 China

## Abstract

Aiming to explore the relationship between the molecular structure and photovoltaic performance, three pyran isomer dyes DO, DM and DP were synthesized and applied as a co-sensitizer with N719 dye in dye-sensitized solar cells (DSCs). These sensitizers were investigated by theoretical calculation, UV-vis absorption spectroscopy and cyclic voltammetry measurement to understand their structure, optical and electrochemical properties. The DSC devices based on N719 and the co-sensitizers were characterized using *I*–*V* tests, incident photon-to-current conversion efficiency and electrochemical impedance spectroscopy measurements. As compared to the standard N719-based DSCs, the co-sensitization system of N719 and DM with the most sterical structure exhibited an enhancement of the power conversion efficiency (PCE) by 18% from 7.60% to 8.96%. Both the short-circuit photocurrent density (*J*_sc_) and open-circuit voltage (*V*_oc_) of the co-sensitized systems were increased resulting from the better maintained N719 dye loading amount on TiO_2_ as well as the prevention of dye aggregation. Co-sensitization of the DO molecule with less steric hindrance reduced the desorbed N719 dye amount by half leading to a decline of the photo-harvesting ability and photocurrent generation in DSCs.

## Abstract 2

This work applied three isomers with different spatial geometries as co-sensitizers to enhance N719-based dye sensitized solar cells.

## Graphical abstract

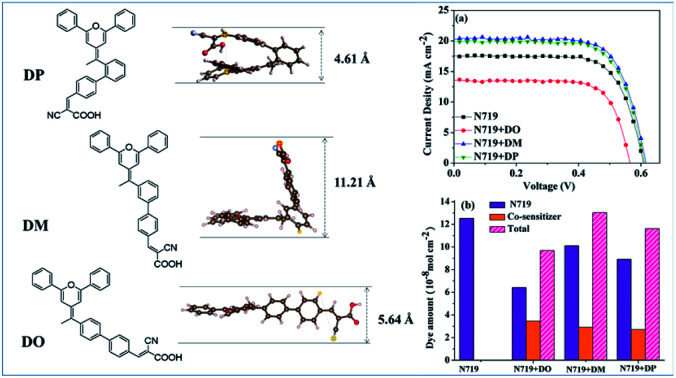

## 1. Introduction

**Fig. 1:**
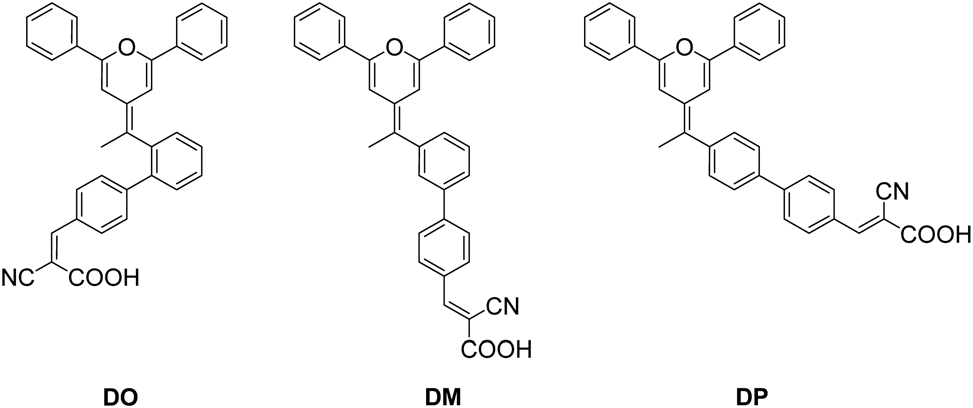
The molecular structure of the three pyran co-sensitizers.

Since being developed by Micheal Grätzel and Brian O'Regan in 1991,^1^ with the advantages of light weight, flexibility and low manufacturing cost, dye-sensitized solar cells (DSCs) have become one of the most promising photovoltaic devices with potential for a wide range of modern indoor applications, such as fifth generation wireless technology and the Internet of Things (IoT).^2–4^ Usually, a DSC is composed of a light-harvesting dye, a semiconducting metal oxide photoanode (such as TiO_2_), a redox electrolyte system and a catalyzing counter cathode. Numerous studies have been performed in the field of DSCs to achieve competitive photovoltaic performance through developing novel materials and promising device structures. In recent years, it has been reported that the power conversion efficiency (PCE) of DSCs has exceeded 14% by application of a silylanchor-based pure organic dye and porphyrin dyes.^5–7^ Utilization of a cobalt-based redox electrolyte instead of the iodide/triiodide system is an efficient method to improve the open-circuit voltage (*V*_oc_),^8–10^ while the enhancement of the short-circuit current (*J*_sc_) is mainly dependent on the dye materials.^11,12^ Ideally, the energy power input process should be completed by a dye that absorbs light energy over an expanded wavelength range to maximize the light-harvest efficiency.^13^ However, it is still challenging to develop a perfect single dye to fulfil extended intensive light absorption that covers wavelengths from ultraviolet to near infrared light. For this reason, the application of multicomponent light-harvesting materials, namely a co-sensitization system, is comparatively practical in DSCs and has received much attention in this field.^14–16^ To date, various kinds of organic dyes have been studied as co-sensitizers and they mainly play the following roles in multi-sensitized DSCs. First, the co-sensitizer complements the photo-absorption gap in an extended wavelength range. This is a similar strategy to that used in another kind of photovoltaic technology, known as organic polymer solar cells (OPVs), where the electron-donor material and electron-acceptor material respond to different wavelength ranges of light energy to optimize the photo-harvesting ability.^17–19^ Kim *et al.*^20^ developed a co-sensitization system consisting of phenothiazine RED dye and squaraine BLUE dye to realize panchromatic light harvesting. The light complementing effect was distinctly reflected in the incident photon-to-current conversion efficiency (IPCE) spectra of the co-sensitized cells. Second, the co-sensitizer enhances the absorption intensity in the same wavelength region. Yao *et al.*^21^ reported co-sensitized cells based on N719 ruthenium dye and dithiofulvenyl-phenothiazine organic dyes. The latter dyes exhibit a relatively higher molar extinction coefficient than N719 within the wavelength range of 320–500 nm and enhanced the *J*_sc_ and IPCE value in the co-sensitized system. Third, the co-sensitizer inhibits main-dye aggregation and thus electron recombination as well as the dark current, as a function of the co-adsorbent,^22^ such as chenodeoxycholic acid (CDCA).^23^ Cao *et al.*^24^ presented co-sensitization of N719 with a series of pyridine-anchored dyes that hindered the aggregation of the main dye N719 to benefit better charge transfer, and the best PCE value achieved from the co-sensitization reached 7%, which is greater than that of the DSCs based on individual N719 (5.43%). In DSC engineering, dye aggregation needs to be avoided by the design of light-harvesting dyes with a sterical structure and the utilization of a co-sensitizer along with the main dye.^25^ The spatial geometric features of the dye and co-sensitizer could hinder the dye aggregates, while also reducing the dye loading amount on the TiO_2_ through blocking the anchoring site. In order to research the factors that have a more important influence on the DSC photovoltaic performance, it is essential to develop suitable co-sensitizers for the progress of this field. Aiming to explore new materials as well as to investigate the relationship between the structure and photovoltaic properties, herein, we present the design, synthesis and characterization of three isomer dyes, DO, DM and DP, with a diphenylpyran donor part, two phenyl rings and a cyanoacrylic acid acceptor moiety. The first phenyl ring combined with the donor group connected to the second one at its *ortho*, *meta* and *para* position, generating three dimensionally diverse structures as shown in Fig. 1. The influence of the three isomer dyes with different sterical structures on N719-based dye sensitized solar cells was investigated using a co-sensitization system. This study showed that the steric structural diversity of the co-sensitizer effects the compatibility between the dyes during the co-sensitization process and an optimized molecular configuration of the co-sensitizer helps to enhance the photovoltaic performance of DSCs through advancing the respective superiority of the dyes.

## 2. Experimental

### 2.1 Materials

Solvents were distilled from appropriate reagents. All reagents were purchased from Sigma-Aldrich, Shanghai Macklin and Shanghai Aladdin Bio-Chem Technology. All reactions were carried out under an inert gas atmosphere.

### 2.2 Instrumentation

^1^H NMR spectra were recorded with a Bruker 600 MHz spectrometer at room temperature and tetramethylsilane (TMS) was applied as an internal reference for calibration. Absorption was recorded through a PerkinElmer Lambda 35 UV-visible spectrometer at room temperature. The redox potentials were measured using a ZAHNER CIMPS photo-electrochemical workstation under an argon atmosphere (supporting electrolyte: 0.1 M (*n*-C_4_H_9_)_4_N-PF_6_, working electrode: glassy carbon electrode, counter electrode: Pt wire, reference electrode: saturated calomel electrodes, SCE).

### 2.3 Synthesis and characterization

Synthesis of tributyl(2,6-diphenyl-4*H*-pyran-4-yl)phosphonium tetrafluoroborate (1a). The synthesis of tributyl(2,6-diphenyl-4*H*-pyran-4-yl)phosphonium tetrafluoroborate (1a) was performed following the literature procedures.^26^

#### 2.3.1 Synthesis of 4-(1-(2-bromophenyl)ethylidene)-2,6-diphenyl-4*H*-pyran (d-*ortho*-2)

Compound 1a (0.76 g, 1.51 mmol) dissolved in 50 mL of anhydrous THF was added to *n*-BuLi in hexane solution (2.83 mL, 4.53 mmol) at 78 °C under argon protection. The solution was stirred at 78 °C for 15 min and 1-(2-bromophenyl)ethanone (0.60 g, 3.02 mmol) dissolved in dry THF (4 mL) was added dropwise. The solution was stirred at 78 °C for 30 min and then moved to room temperature and stirred overnight. After the reaction, the solution was extracted with dichloromethane and the combined organic phase was dried over anhydrous sodium sulfate for 15 min, filtered and evaporated. The product was purified by column chromatography on silica gel (dichloromethane/*n*-hexane, 1 : 2) to give an orange solid (0.13 g). Yield: 20.8%. Mp: 135–138 °C. ^1^H NMR (600 MHz, CDCl_3_) *δ* 7.81 (d, *J* = 6.2 Hz, 2H), 7.66 (d, *J* = 8.0 Hz, 1H), 7.56 (d, *J* = 5.5 Hz, 2H), 7.45 (d, *J* = 6.8 Hz, 2H), 7.41–7.38 (m, 1H), 7.34 (t, *J* = 7.2 Hz, 4H), 7.23 (s, 1H), 7.17 (d, *J* = 7.5 Hz, 1H), 6.57 (s, 1H), 5.91 (s, 1H), 2.10 (s, 3H).

#### 2.3.2 Synthesis of 2′-(1-(2,6-diphenyl-4*H*-pyran-4-ylidene)ethyl)-[1,1′-biphenyl]-4-carbaldehyde (d-*ortho*-1)

d-ortho-2 (0.40 g, 0.97 mmol) was dissolved in toluene (30 mL), and 2.06 g of Na_2_CO_3_ in 10 mL H_2_O and (4-formylphenyl)boronic acid (0.73 g, 4.85 mmol) in ethanolic solution (10 mL) were added. The mixture was deoxygenated under reduced pressure and flushed with argon. Pd(PPh_3_)_4_ (0.11 g) was added and the resulting suspension was heated under reflux overnight. After cooling to room temperature, the mixture was extracted three times by dichloromethane. The combined organic phase was washed twice with brine, dried over anhydrous sodium sulfate for 15 min, filtered and evaporated. The product was purified by column chromatography on silica gel (dichloromethane/*n*-hexane, 1 : 2) to give an orange solid (0.12 g). Yield: 27.6%. Mp: 238–241 °C. ^1^H NMR (600 MHz, CDCl_3_) *δ* 10.00 (s, 1H), 7.85 (d, *J* = 8.2 Hz, 2H), 7.76 (d, *J* = 7.4 Hz, 2H), 7.59 (d, *J* = 7.4 Hz, 2H), 7.56 (d, *J* = 8.1 Hz, 2H), 7.45–7.40 (m, 5H), 7.38 (dd, *J* = 6.9, 4.6 Hz, 3H), 7.35–7.32 (m, 2H), 6.41 (d, *J* = 1.8 Hz, 1H), 6.25 (d, *J* = 1.7 Hz, 1H), 1.73 (s, 3H).

#### 2.3.3 Synthesis of 2-cyano-3-(2′-(1-(2,6-diphenyl-4*H*-pyran-4-ylidene)ethyl)-[1,1′-biphenyl]-4-yl)acrylic acid (DO)

To a stirred solution of compound d-*ortho*-1 (0.10 g, 0.23 mmol) and cyanoacetic acid (0.08 g, 0.92 mmol) in acetonitrile (40 mL) was added piperidine (0.17 mg, 1.94 mmol). The reaction mixture was refluxed under argon for 5 h. After cooling the solution to room temperature and filtering the precipitate, the product was purified by column chromatography on silica gel (dichloromethane/methanol, 6 : 1) to give an orange solid (0.07 g). Yield: 57.8%. Mp: 225–228 °C. ^1^H NMR (600 MHz, DMSO) *δ* 8.00 (s, 1H), 7.89 (dd, *J* = 21.9, 7.8 Hz, 4H), 7.54 (dd, *J* = 18.1, 7.9 Hz, 4H), 7.49–7.38 (m, 10H), 7.32 (s, 1H), 6.71 (s, 1H), 6.11 (s, 1H), 1.86 (s, 3H)

#### 2.3.4 Synthesis of 4-(1-(3-bromophenyl)ethylidene)-2,6-diphenyl-4*H*-pyran (d-*meta*-2)

The reaction proceeded with 1a and 1-(3-bromophenyl)ethenone, following a similar synthesis and purification process to those of compound d-*ortho*-2 and gave a pale yellow solid. Yield: 25.6%. Mp: 103–106 °C. ^1^H NMR (600 MHz, CDCl_3_) *δ* 7.79 (d, *J* = 7.5 Hz, 2H), 7.60 (d, *J* = 7.5 Hz, 2H), 7.49–7.42 (m, 3H), 7.42–7.29 (m, 5H), 7.25–7.21 (m, 2H), 6.53 (s, 1H), 6.46 (s, 1H), 2.13 (s, 3H).

#### 2.3.5 Synthesis of 3′-(1-(2,6-diphenyl-4*H*-pyran-4-ylidene)ethyl)-[1,1′-biphenyl]-4-carbaldehyde (d-*meta*-1)

Similar synthesis and purification to those of compound d-*ortho*-1 gave a pale yellow solid. Yield: 43.8%. Mp: 146–149 °C. ^1^H NMR (600 MHz, DMSO) *δ* 10.02 (s, 1H), 7.97 (d, *J* = 8.3 Hz, 2H), 7.93–7.89 (m, 4H), 7.64 (d, *J* = 8.9 Hz, 2H), 7.56 (d, *J* = 7.4 Hz, 2H), 7.52 (s, 1H), 7.48 (t, *J* = 7.6 Hz, 2H), 7.43 (s, 1H), 7.41–7.37 (m, 3H), 7.34 (s, 1H), 6.82 (d, *J* = 1.9 Hz, 1H), 6.48 (d, *J* = 1.8 Hz, 1H), 2.20 (s, 3H).

#### 2.3.6 Synthesis of 2-cyano-3-(3′-(1-(2,6-diphenyl-4*H*-pyran-4-ylidene)ethyl)-[1,1′-biphenyl]-4-yl)acrylic acid (DM)

Similar synthesis and purification to those of compound DO gave a pale yellow solid. Yield: 58.6%. Mp: 178–181 °C. ^1^H NMR (600 MHz, DMSO) *δ* 8.02 (s, 1H), 7.99 (d, *J* = 7.8 Hz, 2H), 7.94 (d, *J* = 7.5 Hz, 2H), 7.86 (d, *J* = 7.9 Hz, 2H), 7.65 (d, *J* = 12.3 Hz, 2H), 7.59 (d, *J* = 7.4 Hz, 2H), 7.56–7.40 (m, 7H), 7.38 (d, *J* = 5.5 Hz, 2H), 6.85 (s, 1H), 6.53 (s, 1H), 2.23 (s, 3H).

#### 2.3.7 Synthesis of 4-(1-(4-bromophenyl)ethylidene)-2,6-diphenyl-4*H*-pyran (d-*para*-2)

The reaction proceeded with 1a and 1-(4-bromophenyl)ethenone, following similar synthesis and purification to those of compound d-*ortho*-2 and gave a pale yellow solid. Yield: 45.2%. Mp: 162–165 °C. ^1^H NMR (600 MHz, CDCl_3_) *δ* 7.79 (d, *J* = 5.2 Hz, 2H), 7.60 (s, 2H), 7.49 (d, *J* = 8.4 Hz, 2H), 7.47–7.30 (m, 6H), 7.19 (d, *J* = 7.7 Hz, 2H), 6.53 (s, 1H), 6.46 (s, 1H), 2.12 (s, 3H).

#### 2.3.8 Synthesis of 4′-(1-(2,6-diphenyl-4*H*-pyran-4-ylidene)ethyl)-[1,1′-biphenyl]-4-carbaldehyde (d-*para*-1)

Similar synthesis and purification to those of compound d-*ortho*-1 gave a yellow solid. Yield: 37.9%. Mp: 230–233 °C. ^1^H NMR (600 MHz, CDCl_3_) *δ* 9.99 (s, 1H), 7.90 (d, *J* = 7.9 Hz, 2H), 7.75 (d, *J* = 7.9 Hz, 4H), 7.61 (d, *J* = 7.9 Hz, 5H), 7.39 (d, *J* = 6.0 Hz, 5H), 7.32–7.28 (m, 2H), 6.60–6.45 (m, 2H), 2.13 (s, 3H).

#### 2.3.9 Synthesis of 2-cyano-3-(4′-(1-(2,6-diphenyl-4*H*-pyran-4-ylidene)ethyl)-[1,1′-biphenyl]-4-yl)acrylic acid (DP)

Similar synthesis and purification to those of compound DO gave a yellow solid. Yield: 42.8%. Mp: 270–273 °C. ^1^H NMR (600 MHz, DMSO) *δ* 8.01 (d, *J* = 8.2 Hz, 3H), 7.94 (d, *J* = 7.5 Hz, 2H), 7.89 (d, *J* = 8.2 Hz, 2H), 7.82 (d, *J* = 8.2 Hz, 2H), 7.64 (d, *J* = 7.6 Hz, 2H), 7.52 (t, *J* = 7.5 Hz, 2H), 7.48–7.40 (m, 5H), 7.21 (dd, *J* = 41.3, 7.4 Hz, 2H), 6.85 (s, *J* = 1.4 Hz, 1H), 6.60 (s, *J* = 1.2 Hz, 1H), 2.21 (s, 3H).

### 2.4 Preparation, fabrication and measurements of DSCs

F–SnO_2_ (FTO)-coated glass substrates (thickness: 2.2 mm) were cleaned with detergent solution, ultrapure water and ethanol, in order, using an ultrasonic bath, rinsed with water and ethanol, and then dried using N_2_ current. Nanocrystalline TiO_2_ films were prepared using the screen-printing technique with 6 layers of commercially available titanium dioxide paste (Dalian HeptaChroma Solar Tech Co., Ltd., DHS-TPP3, consisting of 20 nm nanoparticles). The film thickness was measured to be 12–14 μm. The films were gradually sintered in a muffle furnace from room temperature to 500 °C and retained for 15 min. TiCl_4_ treatment was carried out with a 40 mM aqueous TiCl_4_ solution at 70 °C for 30 min and washed with H_2_O and EtOH, sequentially. The prepared TiO_2_ electrodes were activated under 80 °C for 30 min before being immersed into the corresponding dye solution. For cocktail co-sensitization, the TiO_2_ electrodes were immersed into a mixture of 0.2 mM EtOH solution of N719 and 0.2 mM CHCl_3_ solution of the diphenylpyran dyes for 24 h. The stepwise co-sensitization was completed through immersion of the electrode in a 0.25 mM EtOH solution of N719 for 15 h and then in a 0.18 mM CHCl_3_ solution of the diphenylpyran dyes for 1 h. Pt-counter electrodes were prepared using the screen-printing technique with 1 layer of commercially available Pt nanocluster paste (Dalian HeptaChroma Solar Tech Co., Ltd., DHS-PtSP) and then sintered at 400 °C for 30 min. The dye-adsorbed TiO_2_ film electrode and Pt-counter electrode were assembled into a sealed sandwich solar cell with a hot-melt Surlyn film (25 μm in thickness) as a spacer between the electrodes. A drop of the electrolyte solution {0.6 M 1,2-dimethyl-3-*n*-propylimidazolium iodide (DMPImI) + 0.1 M LiI + 0.2 M I_2_ + 0.5 M 4-*tert*-butylpyridine (TBP) in acetonitrile} was driven into the cell through the hole in the counter electrode *via* vacuum suction. Finally, the hole was sealed using additional hot-melt Surlyn film covered with a thin glass slide. The prepared dye-sensitized solar cells were illuminated through the conducting glass support with a lightproof black mask with an aperture area of 0.03 cm^2^ to avoid the penetration of diffuse light into the active dye-loaded film (the apparent surface area of the TiO_2_ film electrode was *ca.* 0.36 cm^2^). The performance of the dye-sensitized solar cells was characterized by incident photon-to-current conversion efficiency (IPCE) and photocurrent–voltage (*I*–*V*) measurements. The IPCE measurements were carried out with a ZAHNER CIMPS photo-electrochemical workstation. Photocurrent–voltage was measured by an AAA grade solar simulator (XES-70S1, SAN-EI Electric, Japan) calibrated with a standard photovoltaic cell (AK-200, Konica Minolta Inc.) at 100 mW cm^−2^ using simulated AM 1.5G sunlight.

## 3. Results and discussion

### 3.1 Synthesis and characterization

**Scheme 1:**
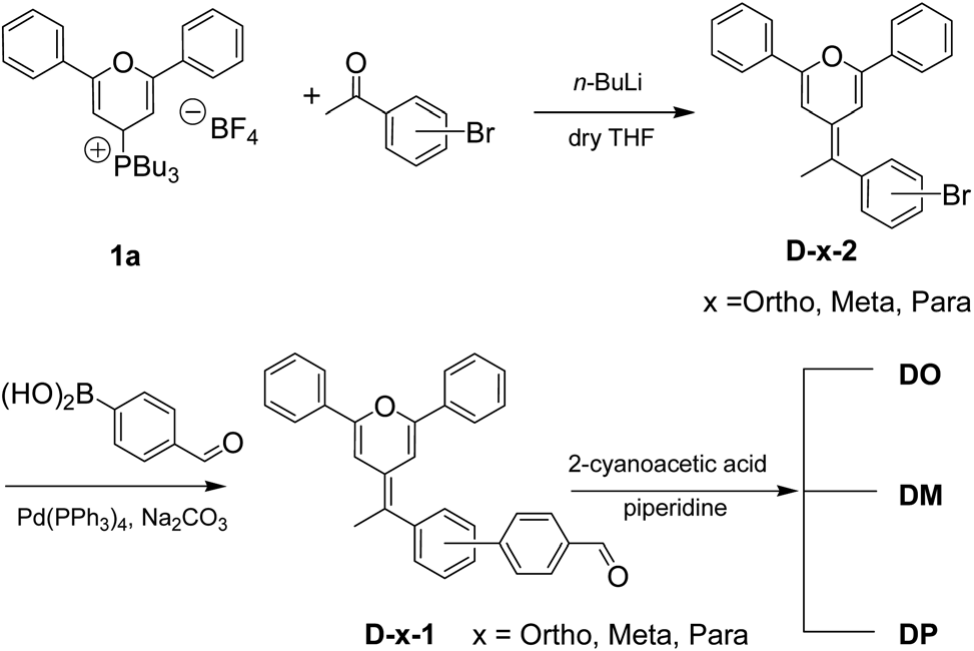
Synthetic route of three diphenylpyran co-sensitizers.

Synthesis of the diphenylpyran dyes (Scheme 1) was completed according to our previous reports.^26–28^ The general synthesis of the diphenylpyran dyes was accomplished in mainly three steps as shown in Scheme 1. Tributyl(2,6-diphenyl-4*H*-pyran-4-yl)phosphonium tetrafluoroborate 1a was prepared following the literature procedures.^26^ The Wittig reaction began with the addition of *n*-butyllithium towards a THF solution of the pyran starting material 1a and completed after reaction with bromoacetophenone, producing D-x-2 (x = *Ortho*, *Meta* and *Para*). Suzuki coupling reaction of the obtained D-x-2 and 4-formylbenzene boric acid occurred to give aldehyde intermediates D-x-1, which were condensed with cyanoacetic acid to give the final dye product DO, DM and DP, respectively. The melting points of the final products and the aldehyde intermediates were also measured.

### 3.2 Theoretical calculations

**Fig. 2:**
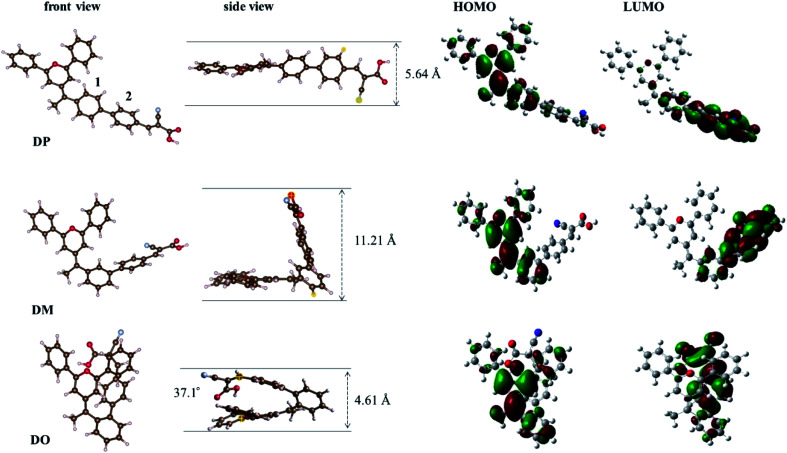
Calculated geometrical structures and electron distribution on the molecular orbitals of dyes DO, DM and DP.

In order to further understand the geometrical configurations and frontier molecular orbitals of the three isomer dyes, theoretical calculations were carried out through density functional theory (DFT) using the B3LYP/6-31G* program with Gaussian 09. The optimized structures of these dye molecules are shown in Fig. 2 and the related data are summarized in Table 1. The DP molecule with the most conjugated structure presents an expanded architecture. Along with phenyl ring 2 adjacent to the cyanoacrylic acid shifting from the *para*-position of phenyl ring 1 near the pyran ring to the *meta*- and *ortho*-position, the patulous structure gradually bends to a folded molecule DO, where phenyl ring 2 constitutes a face-to-face stacking structure with the pyran moiety. The cyanoacrylic acid acceptor group is almost in the same plane as phenyl ring 2 in DM and DP, while it twists with phenyl ring 2 by an angle of 37.1° in DO, turning toward the electron donating pyran ring. Besides, the distance between the oxygen of the carbonyl group and the hydrogen of hydroxyl group gets 0.02 Å larger in DO than in DM and DP. As the structural disparity occurs, the O–H bond length of the hydroxyl group in the DO molecule also elongates from 0.97 Å in DM and DP to 0.98 Å. Furthermore, as shown in Table 2, the melting point *T*_m_ of the dye DO is 13 °C lower than its aldehyde intermediate, while the *T*_m_ of the dye DM and DP is 32 and 40 °C higher than that of their aldehyde forms. These observations imply that there is a strong intramolecular interaction generated between the electron donor pyran ring and the electron accepting moiety in the DO molecule. Along the same axis, the measured lengths of space for the molecules DO, DM and DP are 4.61, 11.21 and 5.64 Å, respectively, suggesting that the DM co-sensitizer should possess a more sterical effect between the dye molecules, while DO rather tends to undergo aggregation in the application of DSCs. The molecular orbital electron distributions of the highest occupied molecular orbital (HOMO) and the lowest unoccupied molecular orbital (LUMO) levels of DO, DM and DP are also shown in Fig. 2 and the corresponding data are listed in Table 1. The HOMO electron density of DM is mainly distributed along the donor moiety and phenyl ring 1, while the HOMO of DO and DP further populates the phenyl ring 2 adjacent to the acceptor moiety. The LUMO electron density of the dye DM is greatly located over the cyanoacrylic acid and π-conjugated spacer, while the electron donating pyran unit of the DO and DP dyes participates in LUMO distribution to a certain extent. These results indicate that light-illumination excited electrons in the DM molecule are capable of transferring from the dye to the TiO_2_ cathode more actively over dye DP and DO.^29^

**Table 1:** Comparison of DFT calculated data of DO, DM and DP

Dye	Dihedral angle^a^ (degree)	O⋯H distance^b^ (Å)	O–H length^c^ (Å)	*E*_HOMO_ (eV)	*E*_LUMO_ (eV)
DO	37.1	2.29	0.98	−4.92	−2.22
DM	0.3	2.27	0.97	−4.85	−2.54
DP	0.2	2.27	0.97	−4.88	−2.54

a Angle between the cyanoacrylic acid and the adjoining phenyl ring 2.

b Distance between the oxygen of the carbonyl group and the hydrogen of hydroxyl group.

c H–O bond length of the cyanoacrylic acid group.

**Table 2:** Photophysical and electrochemical data of the pyran dyes

Dye	*λ*_max_ a (nm)	*ε*_max_ b (M^−1^ cm^−1^)	*λ*_max_ c (nm)	*E*_ox_ d (V)	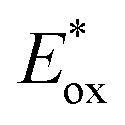 e (V)	*T*_m_ f (°C)	*T*_m_ g (°C)
DO	332	34 800	396	0.92	−1.55	225	238
DM	346	17 500	377	0.86	−1.35	178	146
DP	345	21 900	388	0.88	−1.24	270	230

a Absorption maximum in CHCl_3_ solution.

b Molar coefficient of the maximum absorption in solution.

c Absorption maximum on TiO_2_ film.

d Measured by using cyclic voltammetry in DMF; potentials measured *vs.* SCE were converted to NHE by addition of 0.245 V.

e Calculated from the differences between *E*_ox_ and the absorption edge.

f Melting point of the dyes.

g Melting point of the corresponding aldehyde intermediates.

### 3.3 Spectroelectrochemical properties

**Fig. 3:**
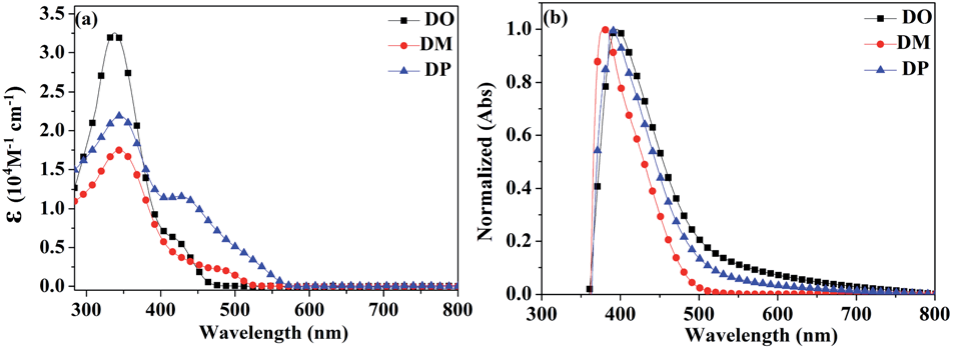
UV-vis absorption spectra of dyes DO, DM and DP (a) in CHCl_3_ solution and (b) on TiO_2_ films (with normalization).

A PerkinElmer Lambda 35 UV-vis spectrophotometer was used to measure the UV-vis absorption spectra of the three dyes in CHCl_3_. The spectra are shown in Fig. 3a and the corresponding spectral data are listed in Table 2. The π–π* transition of the π conjugated system leads to the maximum absorption intensity of dyes DO, DM and DP at 332, 346 and 345 nm, with the corresponding molar absorption coefficient of 34 800, 17 500 and 21 900 M^−1^ cm^−1^, respectively. The absorption bulges of the three dyes in the range of 400–600 nm are associated with intramolecular charge transition (ICT). The TiO_2_ films adsorbed with the dyes were prepared by immersion of TiO_2_ into a 0.3 mM dye solution in CHCl_3_ for 24 h under dark conditions. The normalized UV-vis absorption spectra of DO, DM and DP on TiO_2_ film are shown in Fig. 3b, with absorption maxima at 396, 377 and 388 nm, respectively, which are red-shifted by 64, 31 and 43 nm compared to those in solution.

**Fig. 4:**
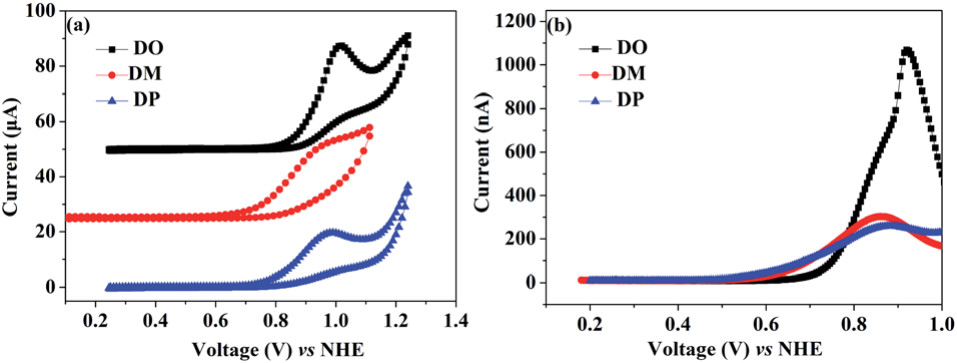
(a) Cyclic voltammograms and (b) differential pulse voltammograms of dyes DO, DM and DP in DMF solutions.

As the red-shift extent follows the trend of the planarity of the three kind of molecules, this bathochromic effect can be ascribed to the dye molecular formation of *J* aggregates on the TiO_2_ surface.^30^ The DM molecule with the most steric configuration gets less red-shifted, suggesting its less aggregation on TiO_2_ compared to the other two dyes. The redox potentials of the dyes were measured in DMF solution by cyclic voltammetry (CV) and differential pulse voltammetry (DPV) in the presence of 0.1 mol L^−1^ tetrabutylammonium hexafluorophosphate as the supporting electrolyte using a scan rate of 100 mV s^−1^ and are listed in Table 2. The CV and DPV curves of the dyes are shown in Fig. 4, respectively. The oxidation potentials *E*_ox_ of DO, DM and DP were observed at 0.92, 0.86 and 0.88 V *vs.* NHE, and the corresponding excited states of oxidation potentials 
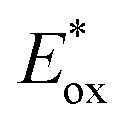
 were located at −1.55, −1.35 and −1.24 V *vs.* NHE, which were calculated from the differences between the ground state oxidation potentials *E*_ox_ and UV-vis absorption onset values. These redox potentials are well-matched with the conduction band of TiO_2_ and the redox potential values of iodide/tri-iodide electrolyte, which is energetically helpful for electron injection and dye-regeneration.^31^

### 3.4 Photovoltaic performance

**Fig. 5:**
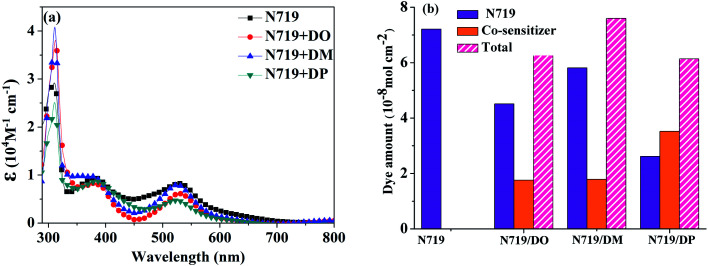
(a) UV-vis absorption spectra of dyes N719, DO, DM and DP in mixed dyeing solutions. (b) Dye amount of individual N719 and the co-sensitizers in mixture solutions.

The current density–voltage (*I*–*V*) characterization of the DSSCs was measured at 100 mW cm^−2^ under simulated AM 1.5G solar light conditions. The DSC based on standard N719 dye exhibited a power conversion efficiency (PCE) of 7.60% with a short current density (*J*_sc_) of 17.52 mA cm^−2^, an open circuit voltage (*V*_oc_) of 608 mV and a fill factor (FF) of 71.36. The DSC devices based on co-sensitization of N719 and the three isomer dyes were fabricated according to our previous report.^28^ When three diphenylpyran dyes were applied as co-sensitizers through a cocktail procedure, the efficiencies of the DSCs dramatically decreased to 3.49%, 4.02% and 2.50% for the DO, DM and DP co-sensitization systems, respectively. As the cocktail solutions were prepared by mixing N719 EtOH solution and co-sensitizers in chloroform solution, an adverse effect on the dye co-sensitization ratios and dye dissolution may lead to insufficient adsorption of N719 on the TiO_2_ electrode. In order to clarify this possibility, the UV-vis absorption spectra of the N719 EtOH solution (1.12 μM) and mixture solution (2.23 μM N719 EtOH solution mixed with 2.23 μM co-sensitizers in chloroform solution with a ratio of 1 : 1) were measured as shown in Fig. 5a. The dye amount of the individual N719 and the co-sensitizers in mixture solutions are presented in Fig. 5b. The molecule content of the dyes in solution were estimated from their absorption intensity and molar extinction coefficient following the literature^32,33^ as described in the ESI† and the data are listed in Table 3. The characteristic light absorption of N719 in a mixture solution decreased obviously in the wavelength range of 400–700 nm, in the order of DM > DO > DP, with the N719 molecule amount of 58.11, 45.06 and 26.24 nmol cm^−2^, respectively. The existence of the total dye amount in the mixture solution is in the order of N719/DM > N719/DO > N719/DP, with the corresponding values of 62.63, 76.03 and 61.48 nmol cm^−2^. This suggested that DM molecules are more compatible with N719 dye than DO and DP. Seeing as the three co-sensitizers exhibit a lack of solubility in EtOH, the DM molecule with more steric effect tends to disperse itself in the solution medium as well as to inhibit the aggregation of N719 molecules to a greater extent than DO and DP, maintaining more N719 molecules in the cocktail solution. This explains why DM presented better PCE in the cocktail co-sensitization than DO and DP. Nevertheless, all three of the co-sensitizers are ineligible to enhance the N719-based DSC through a cocktail procedure, on account of the unfavorable solubility of the sensitizers in mixed solutions.

**Table 3:** Dye amount^a^ in mixture solution and desorbed solutions

Dye		N719 amount (nmol cm^−2^)	Co-sensitizer amount (nmol cm^−2^)	Total (nmol cm^−2^)
N719 only	In mixture solution	72.15	—	72.15
N719/DO		45.06	17.57	62.63
N719/DM		58.11	17.92	76.03
N719/DP		26.24	35.24	61.48
N719 only	In desorbed solution	125.32	—	125.32
N719+DO		64.24	32.61	96.85
N719+DM		101.12	29.24	130.36
N719+DP		89.05	27.17	116.22

a The amounts of N719 and pyran dyes were obtained from the absorbances of the N719 + pyran dye co-sensitization system at 340 nm and 530 nm.

**Fig. 6:**
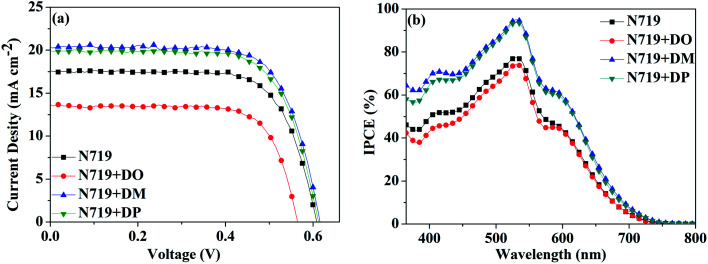
(a) *I*–*V* characterization and (b) IPCE spectra of the DSC based on N719 and stepwise co-sensitization with pyran dyes DO, DM and DP.

**Fig. 7:**
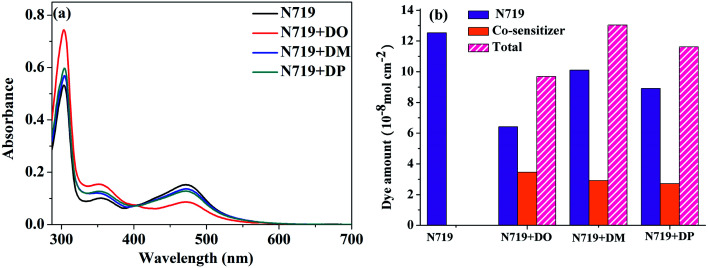
(a) UV-vis absorption spectra of dyes N719, DO, DM and DP desorbed from the co-sensitized TiO_2_ electrode; (b) desorbed dye amount of individual N719 and the co-sensitizers from DSC cells.

**Fig. 8:**
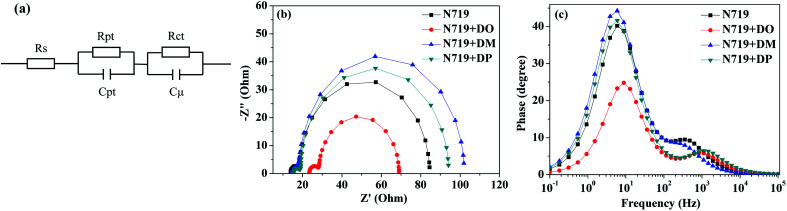
(a) Equivalent circuit for the DSC; (b) Nyquist and (c) Bode plots of the DSCs based on N719 and stepwise co-sensitization with pyran dyes DO, DM and DP.

Subsequently, a stepwise co-sensitization procedure of N719 and the three diphenylpyran dyes was investigated for the purpose of avoiding solubility concerns. *I*–*V* curves for the DSSCs based on the N719 and co-sensitization systems are shown in Fig. 6a, and the corresponding photovoltaic parameters are summarized in Table 4. The DSC based on N719+DM co-sensitization exhibited the highest PCE of 8.96% with a *J*_sc_ of 20.36 mA cm^−2^, *V*_oc_ of 615 mV and FF of 71.52. Under the same fabrication conditions, the cells based on N719+DP co-sensitization gave a *J*_sc_ of 19.94 mA cm^−2^, *V*_oc_ of 611 mV, and FF of 70.68, corresponding to a PCE of 8.62%. Both co-sensitizers enhanced the PCE value by 18% and 13%, due to the increased *J*_sc_ and *V*_oc_. However, when the DO dye was co-sensitized with N719 through a stepwise process, the *J*_sc_ and *V*_oc_ value drop to 14.41 mA cm^−2^ and 568 mV, decreased by 18% and 6.6% as compared to the standard N719 dye. For the purpose of investigating the influence of the three co-sensitizers on the photocurrent generation of the DSC, IPCE measurements for the cells based on the co-sensitization systems were conducted and the spectra are shown in Fig. 6b. The IPCE spectra shift within the wavelength range from 365 to 750 nm in the order of N719+DM > N719+DP > N719 > N719+DO, showing good correlation with tendency of the *J*_sc_ changes. In order to investigate the adsorption amount of the dyes on the electrode, the UV-vis absorption spectra of the dye desorbed solutions (NaOH solution in THF and H_2_O with 1 : 1 ratio) obtained from the N719 and co-sensitized TiO_2_ films were characterized as presented in Fig. 7. When co-sensitizers adsorbed on the TiO_2_ electrodes, the characteristic absorption maximum originating from N719 declined in the order of N719+DM > N719+DP > N719+DO, and the corresponding estimated N719 dye loadings are 101.12, 89.05 and 64.24 nmol cm^−2^, being lower than that of the N719 alone sensitized electrode (125.32 nmol cm^−2^). These phenomena of a decrease in the main dye amount after co-sensitization were also observed in other reported co-sensitization systems.^34–36^ Although co-sensitizers DM and DP presented much less influence on the N719 loading amount, the absorption intensity at 470 nm decreased to half the value when DO adsorbed on TiO_2_ after N719 dye. This result may be explained by the fact that the greater aggregation nature of the co-sensitizer molecules results in rearrangement of the N719 molecules on TiO_2_ during the co-sensitization process^24,37,38^ and the DO molecules with less dimensional barrier competitively reduce the N719 adsorption amount. A similar decrease of N719 dye amount in stepwise co-sensitization has also been reported when the concentration of co-sensitizer increases from 5% to 10%.^34^ This implies that the insufficient adsorption of N719 on the TiO_2_ electrode leads to the dramatic decrease of *J*_sc_ and overall IPCE for the N719+DO system. Next, in the interest of studying the elevated *V*_oc_, electrochemical impedance spectroscopy (EIS) was employed to illustrate the interfacial charge transfer and recombination processes for the DSC. Impedance was performed with an AC amplitude of 10 mV in the frequency range from 10^−1^ Hz to 10^5^ Hz in the dark. The equivalent circuit is presented in Fig. 8a, where *R*_s_ stands for the series resistance; *C*_pt_ and *R*_pt_ represent the interface capacitance and charge transfer resistance at the Pt/electrolyte interface, respectively. *C*_μ_ and *R*_ct_ are the chemical capacitance and electron transport resistance at the TiO_2_/dye/electrolyte interface, respectively. Fig. 8b and c show the Nyquist and Bode plots of the EIS for the DSC based on the co-sensitized systems. The corresponding data are summarized in Table 5. Small and large semicircles were observed in the Nyquist plots, attributed to charge transfer resistance *R*_Pt_ at the Pt/electrolyte interface and electron transport resistance *R*_ct_ at the TiO_2_/dye/electrolyte interface, respectively. The *R*_Pt_ values of all the cells are almost the same due to the same Pt counter electrode and electrolyte being used. The radiuses of the larger semicircles increased in the order of N719+DM > N719+DP > N719 > N719+DO, which is in agreement with the *V*_oc_ values of the DSCs. In the Bode phase plot, the electron lifetime (*τ*_e_) was estimated using the following equation *τ* = 1/(2π*f*_peak_), where *f* is the frequency of the mid-frequency peak. It was obvious that the *f*_peak_ in the EIS Bode plots of lower frequency range increased in the order of N719+DM > N719+DP > N719 > N719+DO, and the *τ*_e_ was enhanced in reverse with the calculated values of 18.05 < 24.88 < 26.99 < 28.43 ms, which was in good agreement with the changing tendencies of the *V*_oc_ values obtained in the *I*–*V* curves. These results indicate that co-sensitization of DM and DP can effectively suppress the injected electron recombination with oxidized triiodide in the electrolyte and thus enhance the open-circuit voltage.

**Table 4:** Photovoltaic performance based on N719 and stepwise co-sensitization with pyran dyes DO, DM and DP

Dye	*J*_sc_ (mA cm^−2^)	*V*_oc_ (mV)	FF (%)	PCE (%)
N719	17.52	608	71.36	7.60
N719+DO	14.41	568	71.04	5.81
N719+DM	20.36	615	71.52	8.96
N719+DP	19.94	611	70.68	8.62

**Table 5:** EIS parameters of DSCs based on N719 and stepwise co-sensitization with pyran dyes DO, DM and DP

Dye	*R*_s_ (Ω)	*R*_pt_ (Ω)	*R*_ct_ (Ω)	*f*_peak_ (Hz)	*τ*_e_ (ms)
N719	13.89	4.33	66.35	6.40	24.88
N719+DO	23.43	4.84	40.75	8.82	18.05
N719+DM	14.68	3.50	83.92	5.60	28.43
N719+DP	15.40	3.58	75.27	5.90	26.99

## 4. Conclusions

Taken together, we studied the influence of the co-sensitizers on the photovoltaic performance of N719-based DSCs, through the development of three pyran isomer dyes DO, DM and DP as well as the investigation of their photophysical properties, adsorbed dye amount and photovoltaic behavior. N719+DM and N719+DP co-sensitization systems presented an enhancement of the energy conversion efficiency of N719-based DSCs by 18% and 13% from 7.60% to 8.96% and 8.62%, respectively, while co-sensitization of DO dye with N719 dropped the DSC efficiency to 5.81%, even though the dye had a larger molar absorption coefficient than the other two isomers. The UV-vis absorption of the solution desorbed from the co-sensitized TiO_2_ electrodes and EIS measurement of the DSC cells showed that the DM molecule with the most sterical structure reveals not only less obstruction of the N719 dye loading amount, but also inhibition of the electron recombination between TiO_2_ and oxidized triiodide in the electrolyte, resulting in improvement of the *J*_sc_ and *V*_oc_. Co-sensitization of the DO molecule with less steric hindrance reduced the desorbed N719 dye amount by half leading to a decline of the photo-harvesting ability and photocurrent generation in DSCs. This study reveals that the prevention of the main dye (N719 and so on) loading amount reduced and the aggregation of the dyes is an important consideration when developing new types of co-sensitizers as well as their application to DSCs. We hope that this work can provide a direction for the development of efficient co-sensitization molecules to achieve enhanced photovoltaic performance. The co-sensitizing conditions for the main dye and co-sensitizers, such as the soaking time, concentration and solvent-dependence, will also influence the dye aggregation behavior, photophysical properties and DSC performance of the dyes. Further investigation of these parameters in co-sensitization is in progress.

## Conflicts of interest

There are no conflicts to declare.

## Supplementary Material

RA-010-D0RA08276G-s001
