# Supplementary material for: The influence of three diphenylpyran isomer co-sensitizers with different sterical structures on N719-based dye sensitized solar cells
Source: RSC Adv. 2020 Dec 7;10(71):43290–8. doi: 10.1039/d0ra08276g (PMC9058243; doi:10.1039/d0ra08276g)
Supplement: RA-010-D0RA08276G-s001 [file RA-010-D0RA08276G-s001.pdf]

Electronic Supplementary Material (ESI) for RSC Advances.
This journal is © The Royal Society of Chemistry 2020

Supporting Information

The influence of three diphenylpyran isomer co-sensitizers with different sterical structure on N719-based dye sensitized solar cells

Xinxin Wang, [ab] Xiuhua Hang,[ab] Altan Bolag, [*a] Wu Yun, [*b] Tana Bao,[a] Jun Ning,[a] Hexig Alata,[a] Tegus Ojiyed[a]

[a]*Inner Mongolia Key Laboratory for Physics and Chemistry of Functional Materials, College of Physics and Electronic Information, Inner Mongolia Normal University, No 81 Zhaowuda Road, Saihan district, Hohhot 010022, China*

[b]*Inner Mongolia Key Laboratory for Environmental Chemistry, College of Chemistry and Environmental Science Inner Mongolia Normal University, No 81 Zhaowuda Road, Saihan district, Hohhot 010022, China*

**Corresponding author: altan.bolag@imnu.edu.cn*

1. Measurement of dye adsorbed amounts

The **N719** and pyran dye co-sensitized electrodes (0.42 cm^2) were immersed into a TiO_2-desorption solution (0.1 mol L^{-1} NaOH, THF / H_2O=1 : 1), leading to desorption of the dye molecules.[1] The UV-vis absorption spectra of the solutions were measured and the adsorption capacity Γ (mol/cm^2) can be calculated by following equations.

$A = \varepsilon bc$ (1)

$n = cv$ (2)

$\Gamma = n/S$ (3)

Where A is the absorbance, ε is the molar absorption coefficient (L/mol·cm), b is the colorimetric plate width (1cm), c is the concentration of the dye solution (mol/L), n is the amount of substances in the sample solution, v is the volume of the sample solution (L), S is the TiO_2 electrode area (0.42 cm^2).

The concentrations of **N719** and pyran dyes (shown as $C_{N719}$and $C_{pyran\ dye}$) were obtained from the absorbances of the **N719** + pyran dye co-sensitization system at 340 nm and 530 nm by using following equations:

$A'_{at\ 340\ nm} = \varepsilon'_{N719}[C_{N719}] + \varepsilon'_{pyran\ dye}[C_{pyran\ dye}]$ (4)

$A''_{at\ 530\ nm} = \varepsilon''_{N719}[C_{N719}] + \varepsilon''_{pyran\ dye}[C_{pyran\ dye}]$ (5)

where A' and A'' are the absorbances at 340 nm and 530 nm, ε'_{N719} and ε''_{N719} are the molar absorption coefficients of N719 at 340 nm and 530 nm; $\varepsilon'_{pyran\ dye}$ and $\varepsilon''_{pyran\ dye}$ are the molar absorption coefficients of pyran dye at 340 nm and 530 nm, respectively.

2. Photovoltaic performance of DSCs based on individual dye DO, DM and DP

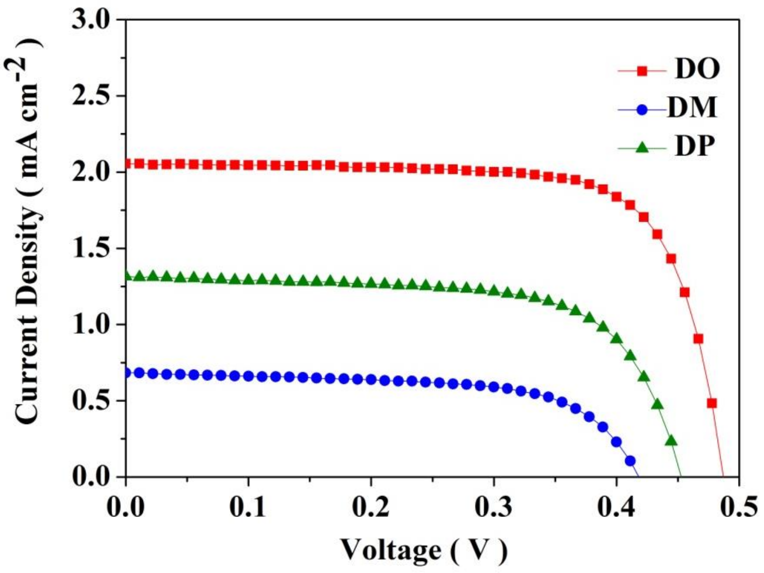

Fig. S1. I-V characterization of the DSC based on pyran dyes **DO**, **DM** and **DP**

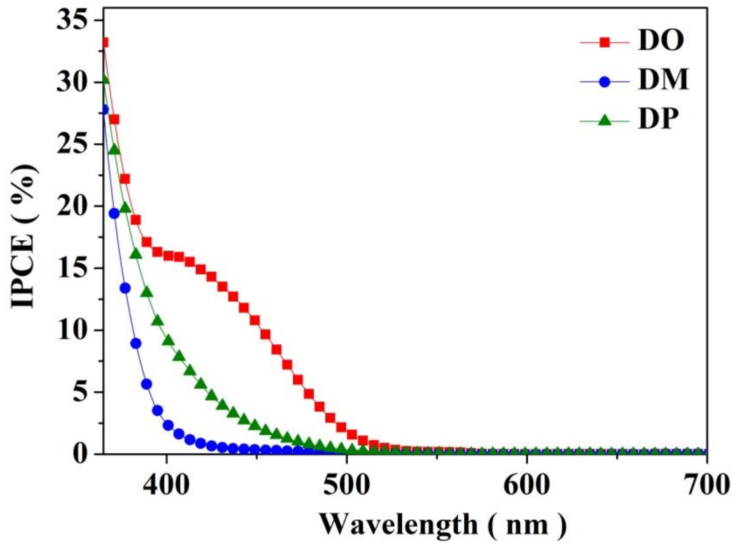

Fig. S2. IPCE spectra of the DSC based on pyran dyes **DO**, **DM** and **DP**

3. **Comparison of UV-vis absorption of the dyes and normalized IPCE spectra of stepwise co-sensitized systems**

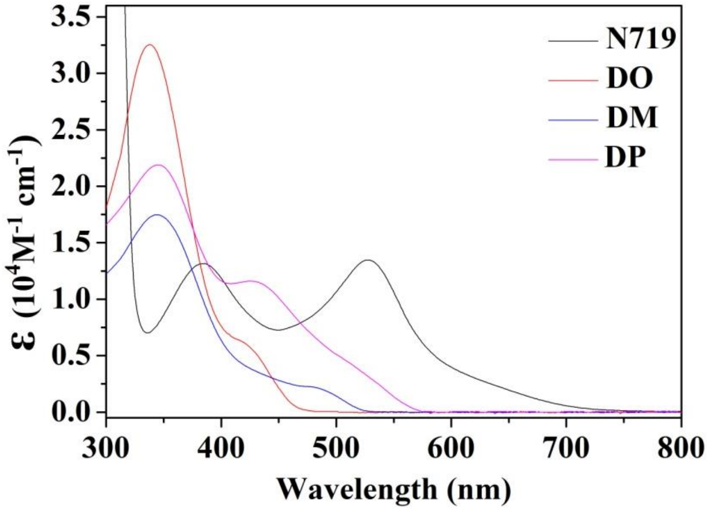

Fig. S3. UV-vis absorption spectra of dyes **DO**, **DM** and **DP,** in comparison with that of **N719**

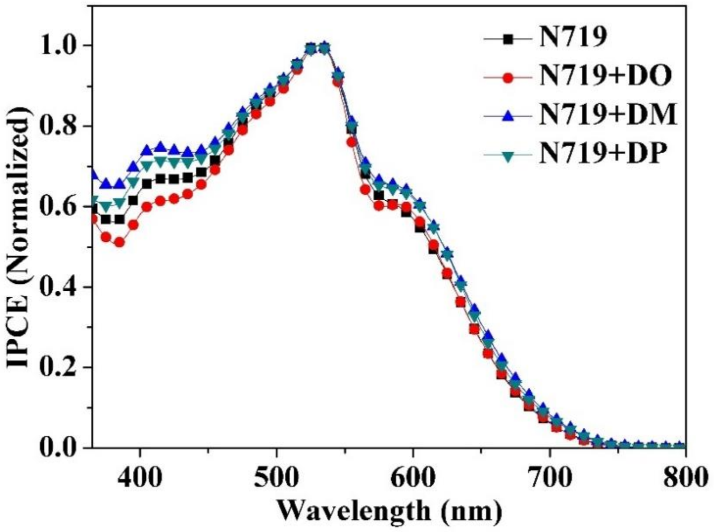

Fig. S4. Normalized IPCE spectra of the DSC based on **N719** and stepwise co-sensitization with pyran dyes **DO**, **DM** and **DP**

4. Comparison of integrated photocurrents from IPCE spectra of stepwise co-sensitized systems

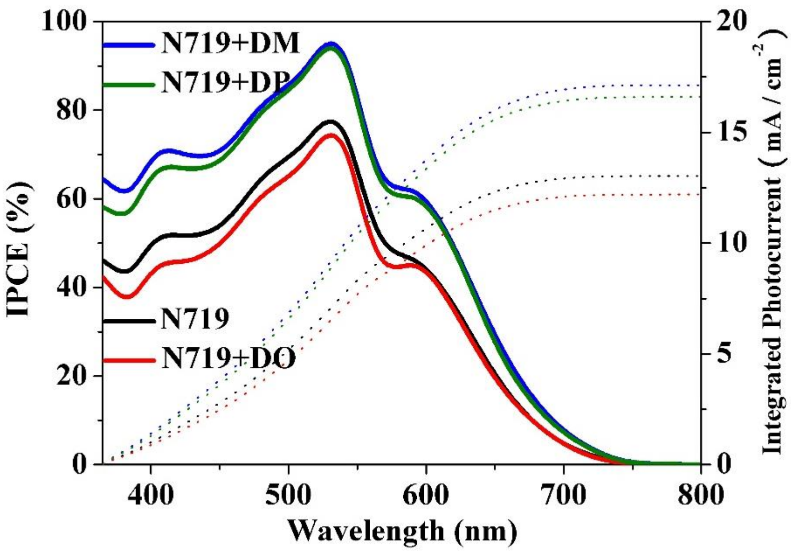

Fig. S5. The IPCE and overlap integral of photocurrent irradiance of the co-sensitization system

5. Comparison of Light-harvesting efficiency (LHE)[2] spectra of stepwise co-sensitized systems

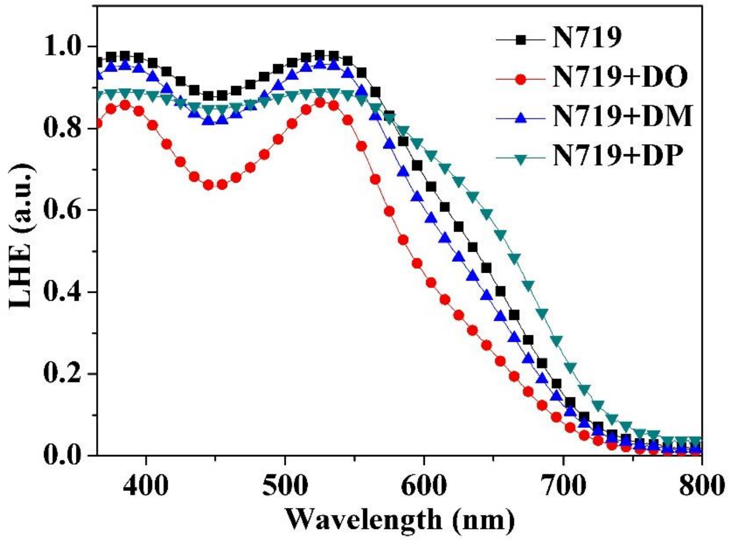

Fig. S6. Light-harvesting efficiency LHE spectra of the DSC based on **N719** and stepwise co-sensitization with pyran dyes **DO**, **DM** and **DP**

Note:

The IPCE value before 365 nm were not measured due to parameter limits of our photo-electrochemical workstation, the *J*sc values of the overlap integral are lower than the *J*sc measured in IV characterizations. However, the trends of the integrated *J*sc shift are in accord with the *J*sc obtained from the J-V plot, being presented in order of **N719**+**DM**> **N719**+**DP**>**N719**> **N719**+**DO**.

References:

1. H.-L. Jia, M.-D. Zhang, Z.-M. Ju, H.-G. Zheng and X.-H. Ju, *J. Mater. Chem. A*, 2015, **3**, 14809-14816
2. M. K. Nazeeruddin, A. Kay, I. Rodicio, R. Humphry-Baker, E. Muller, P. Liska, N. Vlachopoulos, and M. Gratzel, *J. Am. Chem. Soc.*, 1993, **115**, 6382-6390
